# A Systematic Literature Review of the Factors Influencing Hearing Protection Device Usage among Industrial Workers

**DOI:** 10.3390/ijerph20042934

**Published:** 2023-02-08

**Authors:** Nur Syafiqah Fauzan, Ezrin Hani Sukadarin, Mirta Widia, Irianto Irianto, Ihwan Ghazali

**Affiliations:** 1 Faculty of Industrial Sciences and Technology, University Malaysia Pahang, Lebuh Persiaran Tun Khalil Yaakob, Kuantan 26300, Pahang, Malaysia; 2 Department General Education, Faculty of Resilience, Rabdan Academy, Abu Dhabi P.O. Box 114646, United Arab Emirates; 3 Faculty of Mechanical and Manufacturing Engineering Technology, Universiti Teknikal Malaysia Melaka, Hang Tuah Jaya, Durian Tunggal 76100, Malaysia

**Keywords:** hearing protection device, factors, industrial workers, preferred reporting items for systematic, systematic literature review

## Abstract

This systematic literature review (SLR) aims to determine the factors influencing the use of hearing protection devices (HPDs) among industrial workers. This study was guided by the PRISMA Statement (Preferred Reporting Items for Systematic reviews and Meta-Analyses) review method, and four databases comprising Scopus, Science Direct, PubMed, Wiley Online Library, and Google Scholar were employed. A total of 196 articles were identified, and 28 studies on the factors associated with HPD use among industrial workers from 2006 to 2021 met the inclusion criteria. Resultantly, five main themes emerged from this review: sociodemographic (29%), interpersonal influences (18%), situational influences (18%), cognitive-perceptual (29%), and health-promoting behavior (6%) associated with HPD use among industrial workers. A total of 17 sub-themes were identified, including age, gender, educational level, noise level, working experience, social models, interpersonal support, social norms, safety climate, training, organizational support, perceived barrier, perceived susceptibility, perceived severity, perceived benefit, self-efficacy, and cues to action. The significant factors influencing workers to use HPDs are sociodemographic, interpersonal influences, situational influences, and health-promoting behavior. Future studies should focus on the cues to action toward human behavior influencing the use of HPDs, workers’ health status, and comorbidities of hearing loss. Therefore, this systematic study gives valuable reference resources for up-and-coming researchers as well as new knowledge to expert professionals and academics in various industries.

## 1. Introduction

Hearing protection devices (HPDs) are generally used in the workplace to prevent hearing loss caused by loud noise [1]. The device should be worn correctly and consistently while working in a noisy environment. Currently, HPDs are popular globally, and several studies have focused on developing the most effective hearing conservation program (EHCP) [2]. The EHCP aims to minimize the risk of occupational hearing loss, preserve and protect hearing, and provide workers with relevant knowledge and safeguards. Wearing HPDs is a part of EHCP whereby the employer must supply the device when engineering and administrative noise control measures do not reduce the noise exposure below the Noise Exposure Limit (NEL)—as specified in Occupational Safety and Health (Noise Exposure) Regulations 2019 [3]. Most employers choose a hearing protector as part of their ECHP to prevent noise-induced hearing loss (NIHL) [4].

Numerous occupational limits for noise have been established by governmental agencies and non-governmental groups [5]. Currently, the United States Occupational Safety and Health Administration (US OSHA) enforces a permissible exposure limit (PEL) of 90 dBA for eight hours, time-weighted average (TWA), with a 5 dBA time-intensity exchange rate (ER) [6]. Most countries with enforceable occupational exposure limits for noise have chosen to use an exposure limit of 85 dBA with a more protective 3 dBA exchange rate [5]. Moreover, the new Malaysian regulation on the noise exposure limit has also changed to 85 dBA as specified in the Occupational Safety and Health (Noise Exposure) Regulations 2019 [3]. The changes to a stricter noise exposure limit were due to increasing NIHL among industrial workers year on year [7]. Furthermore, the 85 dBA noise exposure limit is similar in other countries, such as Australia [8], New Zealand [9], and China [10]. Meanwhile, Canada and Europe permitted a noise exposure limit of 8 h at 87 dBA, which is less strict compared to Australia, New Zealand, China, and Malaysia. Nevertheless, this permitted noise exposure limit (87 dBA) appears more stringent compared to the limit in the United States at 90 dBA [11].

The World Health Organisation (WHO) projected hearing loss as one of the top 10 causes of disease burden in high- and middle-income countries by 2030 (moving up at least three places since the 2002 rankings) [12]. Furthermore, the WHO estimated that the number of people with disabling hearing loss (DHL) will be more than 900 million at global and regional levels by 2050 [13]. In Malaysia, occupational noise-related hearing disorders were the most prevalent among all occupational poisoning and disease cases between 2016 and 2019 [7]. Further, the Department of Occupational Safety and Health Malaysia reported in 2019 that the highest percentage of confirmed occupational poisoning and disease cases were in the manufacturing sector [7]. A statistically significant relationship between personal noise exposure level and the prevalence of psychological health symptoms among construction workers was also reported [14]. The researchers reported a significantly high prevalence of psychological health symptoms among machine operators. 

The main research question guiding this systematic review is: what are the factors influencing HPD use among industrial workers? This topic has been investigated in several studies considering the significance of HPDs as part of occupational safety and hazard [15]. This review will contribute to the existing body of knowledge and the literature. Specifically, this review aims to determine the factors (read factors) influencing the use of HPD. 

## 2. Methodology

The primary method used to retrieve relevant articles on HPD use among industrial workers was the Preferred Reporting Items for Systematic reviews and Meta-Analyses (PRISMA) Statement. The PRISMA method assists researchers in identifying and screening for eligibility criteria and data abstraction for analysis. This method also assisted the current researchers in defining the research question, identifying the inclusion and exclusion criteria, and examining the large database in a defined time [16].

The PRISMA Statement allows for rigorous search terms related to HPD use amongst the target population in this study, namely industrial workers. The importance of the methodology lies in the fact that it assisted the reviewers in carefully identifying and selecting the factors influencing industrial workers to wear and comply with the use of HPD voluntarily.

The research question for this study was formulated based on PICo [Population or Problem (P), Interest (I), and Context (Co)], which is a research question development tool. This tool was used to develop three main aspects related to the focus of the review: industrial workers, hearing protection devices, and factors. This enabled the researchers to develop the research question, “What are the factors influencing HPD use among industrial workers?”.

### 2.1. Identification Phase

The systematic review process comprised three stages: identification, screening, and eligibility. The first stage entailed the identification of keywords, followed by the process of searching for related words. Search strings on Scopus and Web of Science databases were used to determine all the relevant keywords, as shown in Table 1. The Scopus database efficiently provided relevant and authoritative research articles and access to reliable data, metrics, and analytical tools. It is a unique database that combines a comprehensive, expertly curated abstract and citation database with enhanced data and linked scholarly literature across diverse research areas (https://www.elsevier.com/solutions/scopus accessed on 5 December 2022). The Web of Science database contains all the disciplines and regions that facilitated the retrieval of suitable and relevant journals through its Master Journal List (https://mjl.clarivate.com/home accessed on 5 December 2022).

**Figure 1:**
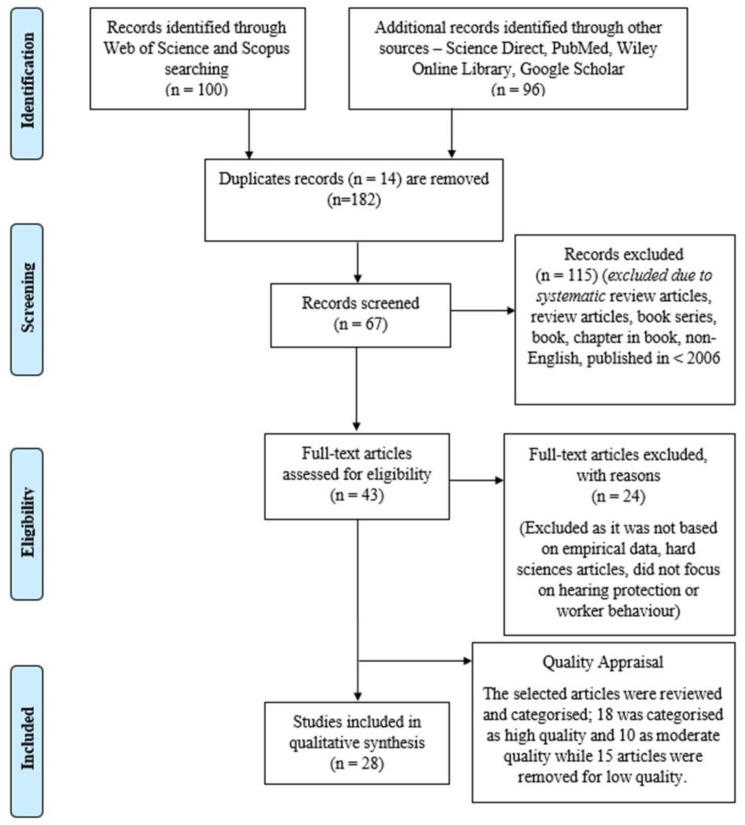
Flow diagram of the study [20,21].

A total of 50 articles each were successfully retrieved from the Scopus database and the Web of Science (WOS). A manual search based on similar keywords was later conducted in the other leading databases, such as ScienceDirect, PubMed, and Wiley Online Library. Meanwhile, Google Scholar was used as a supporting database [17,18,19]. A combination of keywords and related words was used, such as “hearing protection device”, “hearing protector”, “factor”, “aspect”, “cause”, “industrial worker”, and “industrial employee”. All the keywords were selected via functions of phrase searching and the Boolean operator (OR, AND). The search process in Scopus and the Web of Science database produced 100 articles, as shown in Figure 1.

### 2.2. Screening Phase

All 196 selected articles were screened based on the sorting function available in the database. The screening process was based on document type and year of publication. The articles were screened based on specific inclusion and exclusion criteria. First, only articles and conference proceedings were selected, effectively excluding review articles, book series, book chapters, and books. Second, non-English publications were excluded to avoid difficulty and confusion in translating the content of the selected article or conference paper. Therefore, only selected articles and conference proceedings published in English met the current study criteria. Third, a period of 15 years was selected (between 2006 and 2021), an adequate period to assess the evolution of the study and related publications according to the research question and selected keywords. In addition, the selected publication timeline was based on the total number of related publications retrieved and to be reviewed. The presented data results were taken from the selected previous articles within the targeted years. A longer publication timeline was needed as there was a limited number of articles, and many research questions were not answered [22]. Thereafter, the 14 duplicated articles were removed. The inclusion and exclusion criteria are shown in Table 2. 

### 2.3. Eligibility

A total of 24 articles were eliminated upon checking for eligibility. These articles were removed for being irrelevant to the research focus as they lacked empirical data, and hard science articles did not focus on hearing protection or worker behavior. The articles that met the inclusion criteria were reviewed based on their title and abstract. The last stage of review led to a final selection of 43 articles, which were included in the qualitative analysis (see Figure 1).

### 2.4. Quality Appraisal

The selected 43 articles were assessed, appraised, and analyzed for quality. The remaining articles were presented to two experts to ensure the quality of the article’s contents [23]. The articles were ranked into three quality categories: high, moderate, and low. Thereafter, the high and moderate articles were selected for review. The reviewers focused on the methodology of the articles to determine their quality, thus leading to a final selection of 28 articles (10 articles were ranked as moderate and 18 as high), while 15 articles were removed due to low quality.

### 2.5. Data Abstraction and Analysis

The review used a qualitative technique of mixed-method studies (qualitative + quantitative + mixed-method studies). The selected articles were analyzed by focusing on the studies related to the research question. Thematic analysis was performed to identify the themes and sub-themes. The abstract was analyzed before data were extracted, followed by a discussion of the selected articles to identify and develop relevant themes and sub-themes. Thematic analysis is the most suitable method to synthesize a mixed research (integrative) design [24].

The first step in the thematic analysis is to generate themes and sub-themes. Thematic analysis discovers themes and subthemes based on activities, such as recognizing patterns and themes, clustering, counting, noting similarities, and relating the collected data [25]. The authors first attempted to identify patterns that emerged from the selected 28 articles. Any similar data from the abstraction process were pooled into five groups (which were identified during data analysis). The authors later re-examined the five groups and identified 17 sub-groups. All the groups and sub-groups were re-examined to ensure data utility and accuracy. The groups and sub-groups were renamed as themes and sub-themes. 

This technique was used to produce themes in a group consisting of the corresponding author and co-authors, along with the discovered themes. The researchers examined any discrepancies, thoughts, puzzles, or concepts that could be related to the data interpretation during the theme development. This process persisted until the researchers reached an agreement on the adjustment of the developed themes and sub-themes. The themes and sub-themes were evaluated by two experts who endorsed them as suitable for the review.

## 3. Result

### Background of Selected Articles

The thematic analysis led to five themes: sociodemographic, situational, interpersonal, cognitive-perceptual, and health-promoting behavior. A further 17 sub-themes emerged; 12 studies were conducted in the United States, three in Canada, three in Thailand, two in Brazil, and one conducted in Malaysia, UAE, China, Portugal, Belgium, Kenya, Iran, and New Zealand. Four articles were published in 2016, three in 2019, 2015, and 2010, two in 2017, 2013, 2012, 2009, and 2008, and one in 2021, 2018, 2011, 2007, and 2006. 

**Figure 2:**
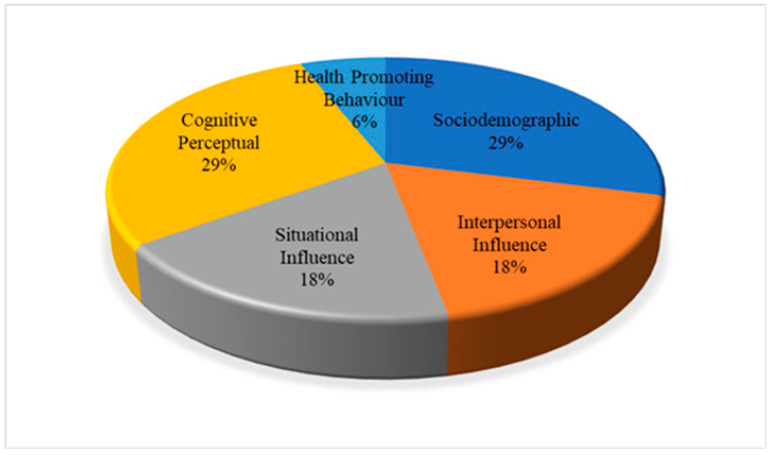
Percentage distribution of themes.

**Figure 3:**
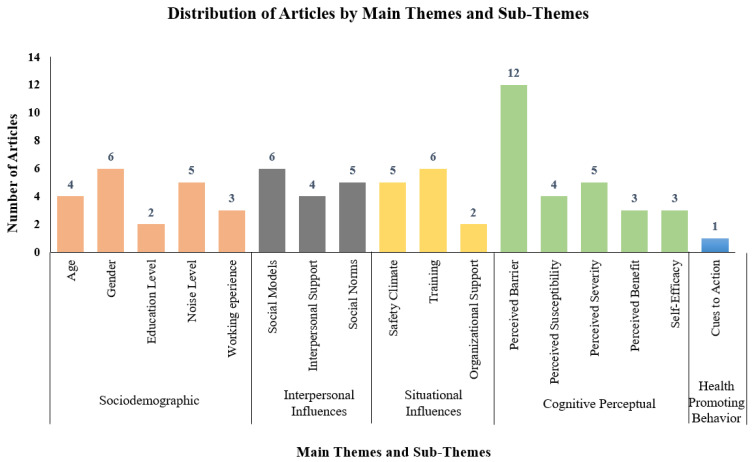
Distribution of articles by main themes and sub-themes in the SLR study (databases accessed were Scopus, Web of Science (WOS), ScienceDirect, PubMed, Wiley Online Library, and Google Scholar) between 2006 and 2021.

Table 3 contains the list of the articles as well as their authors, country, year of publication from 2006 until 2021, themes, and sub-themes. Figure 2 provides the percentage distribution of themes, while Figure 3 shows the number of published papers based on themes, such as sociodemographic, interpersonal influences, situational influences, cognitive-perceptual, and health-promoting behavior from 2006 until 2021, according to the data obtained from Scopus, WOS, ScienceDirect, PubMed, Wiley Online Library, and Google Scholar. 

Figure 2 depicts that the sociodemographic and cognitive-perceptual themes accounted for 29%. Meanwhile, the main themes encompassing interpersonal and situational influences accounted for 18% of the themes. Lastly, the health-promoting behavior theme represents about 6% of the 17 sub-themes.

Figure 3 depicts the distribution of articles by main themes and sub-themes based on thematic analysis. The number of published articles was based on themes and sub-themes. The main themes were cognitive-perceptual (27 studies), sociodemographic (20 studies), interpersonal influences (15 studies), situational influences (13 studies), and health-promoting behavior (one study). The perceived barrier was the most common subtheme (12 studies), whereas gender, social models, and training accounted for the second most important subthemes (six studies each). The health-promoting behavior theme was found in only one study. 

## 4. Discussion

### 4.1. Sociodemographic Characteristics

The level of use and compliance with wearing HPD was influenced by personal factors, such as education, age, and work experience [41]. Additionally, young adult workers were more willing to use HPD as they tended to listen to their employer’s instructions. Young adult workers also demonstrated greater knowledge of occupational hazards regarding the adverse effects of excessive noise exposure, namely the risk of hearing loss [33,48]. Nevertheless, there is still a shortage of empirical evidence on exposure to noise and HPD use among young adults in the United States despite more than 11 million individuals suffering from NIHL [50]. Several previous studies have suggested that as workers get older, they are more willing to wear HPDs [41,42]. Age was also a significant predictor for interpersonal modeling and situational influences on HPD use [42]. Therefore, there are conflicting results regarding the influence of age on the use of HPD among workers. These differences could be due to methodological discrepancies, such as the diversity of industries and jobs within the population under study [39]. 

Moreover, the organizational culture in the workplace would influence the use of HPD. Thus, workers should be encouraged, and the usage of HPD in the workplace should be closely monitored. Therefore, the need for expert supervision and the application of regulatory standards in these industries is unavoidable [41]. 

In terms of gender, female workers have a greater tendency toward HPD use [27,39,48]. Furthermore, female workers are better and more committed supervisors, adhere to safety rules, and have greater safety knowledge [39]. Nonetheless, several earlier studies claimed that consistent use of HPDs is higher among male workers [32,33]. In addition, female workers were found to be negligent, adopting a lackadaisical approach when exposed to noise at their workplace [39]. In contrast, the use of hearing protection does not differ significantly between men and women [51]. Overall, HPD use is multifactorial, and the predictors cannot be reduced to singular factors independently. In other words, other confounding factors could influence the use of HPD among workers. Past researchers mentioned that hearing protection use had been linked to a variety of factors, including work-related factors, such as the use of other safety equipment and personal traits [2]. If these confounding factors vary by company, they could contribute to the observed difference in HPD use. 

Workers’ educational level may also affect their tendency to use HPD [30,41]. Workers with higher levels of education have a greater propensity to wear HPD than those who only attended primary and secondary educational levels [41]. Meanwhile, there was no association between the use of HPDs and the level of education [35]. Higher education enhances problem-solving abilities, cognitive skills, and access to resources that motivate workers to maintain good health practices at the workplace [52]. Hence, safety training (as an initiative to educate workers) is vital for employers to promote awareness among their employees on the importance of safe workplace practices, especially among those with low educational levels [52]. 

Work experience and noise exposure levels can affect workers’ compliance with wearing HPD [2,41,46]. Moreover, exposure to high levels of noise at the workplace may influence workers to wear HPD [34,37]. Past researchers also noted that intermittent noise exposure reduces the worker’s motivation to use HPD compared to continuous noise [3]. Wearing HPDs might also be the most effective approach to addressing exposure to higher ambient noise levels for extended periods [53]. In addition, exposure to lower noise levels creates a false sense of security. Resultantly, risk perception is better in organizations with higher levels of exposure [2]. Moreover, the greater the perception of the health danger posed by noise in the workplace, the more likely workers will adopt safe work positions, such as wearing HPDs [48].

More experienced workers would significantly influence a higher rate of continuous hearing protection use [2]. For example, a higher rate of HPD uses among Canadian workers who are experienced in working in a noisy work environment [46]. The level of HPD use among Iranian carpet-weaving workers also increased as the workers gained more work experience [41]. Meanwhile, employment at multiple job sites and the likelihood of being self-employed were the factors influencing hearing protector usage among construction workers [54]. Contrarily, the use of hearing protectors decreased with increasing age and seniority [55]. Therefore, awareness among workers is crucial to encourage workers to wear hearing protectors while working in a noisy work area. Workers’ age and working experience should also be considered if an organization plans to conduct conservation programs, such as occupational safety promotion and injury prevention [47]. Thus, noise-induced hearing loss symptoms can be prevented to protect workers’ health and improve their well-being.

### 4.2. Interpersonal Influences 

A unique work culture arises from observational learning and support from others, such as co-workers and supervisors. The importance of the interpersonal influence on HPD use and its role in inducing behavioral change toward HPD use among workers have been reported in several studies [4,36,37,45]. Interpersonal influence consists of a social model, interpersonal support, and social norms. The social model refers to the extent to which workers perceive that other co-workers, family members, and managers will use the HPD. Moreover, interpersonal support refers to the encouragement from co-workers, family, and supervisors to use HPD, while social norms emphasize the beliefs of co-workers, family members, and managers that they should wear the HPD [37,40].

Social norm was a significant predictor of greater personal protective equipment (PPE) use among workers [40]. Therefore, a good attitude among co-workers and supervisors towards HPD use would increase workers’ safety and health. In addition, a high level of management support may beneficially influence workers’ health and safety behaviors [52]. Meanwhile, ‘peer mentality’ among workers, whereby some may find wearing HPD ridiculous and anti-social, was reported in [36]; in addition, such workers may discourage their peers from using HPD as they do not value its importance [27,36].

The propensity for workers to use HPD is higher as the score of social models increases [34]. Co-worker modeling, supervisor support, and supervisor modeling are important elements in the interpersonal influence that led to workers’ decision to use hearing protectors [31]. The supervisor’s role is important in preventing workplace injuries, providing information about workplace safety, and establishing clear rules to prevent workers from occupational disease, which positively influences their HPD use [39]. Therefore, HPD-use-related attitudes and beliefs become more positive over time [42].

Interpersonal support among Chinese migrant workers was significantly associated with PPE after controlling for individual demographic characteristics [40]. Likewise, co-workers could mutually encourage and motivate each other towards adopting positive behaviors regarding HPD use if they support each other and remain mindful of safety practices [36]. Other researchers posited that family and peers influence HPD use, which leads to behavioral change. For example, preservation of hearing to maintain a good quality of life was reported as one of the main reasons for wearing HPDs [4,31,36]. 

Other studies have highlighted interpersonal influence in behavioral change related to HPD use [4,36,37,45]. Worker peer, non-work peer, supervisor influence, and family influence play an important role in influencing workers’ attitudes and behavior toward HPD use. Interpersonal models, norms, and support might affect workers more than other factors, especially those who work with other staff and live with their families [40]. A combination of peer and management support or modeling can create a conducive safety culture [56]. Management and co-workers play an important role in maintaining good occupational safety practices. Direct and indirect interactions play essential functions in maintaining individuals’ health and behavior, namely interpersonal level, social networks, and provision of social support like friends and family [56,57]. Interventions incorporating social modeling and interpersonal support by the management or company could also promote employee compliance with wearing HPDs by influencing their behavior and attitude towards HPD [37]. 

### 4.3. Situational Influences

A good hearing conservation practice among workers is directly related to the safety climate at the workplace [2]. Positive relationships have also been reported between an increase in safety climate and HPD use [15,28,30]. The worker’s motivation and perception of occupational hazards and workplace environment play a crucial role in influencing HPD use [28,30]. Nevertheless, there is an inverse association between co-worker safety climate and types of PPE use, such as hearing protection, dust masks, general equipment, and fall protection [38]. Workers are less likely to utilize PPE if they believe it will not reduce the health risks associated with workplace exposures. Risk-takers will pick both unsafe work surroundings and risky behaviors, such as not wearing PPE, thus resulting in a link between the two elements even when no causal relationship exists [58]. Furthermore, the lack of qualified labor, a tight project timetable, and a decreased priority for safety would all result from unsupportive industrial norms in the construction industry [59]. In addition, their participation in the implementation of the safety program would be limited due to a lack of skilled staff.

Employer interventions have been shown to influence workers’ behavior, thereby motivating them to wear HPDs at their workplace [42]. Training is one of the popular intervention programs that could lead to positive behavioral change among workers [4,42,47]. Regular training at workplaces on noise and hearing protection tools also encourages workers to wear HPD [36]. Additionally, regular training increases workers’ awareness of issues related to hearing protection. Training also indirectly affects risk perception and behavior on PPE use [48]. In summary, the PPE use rate among workers results from instruction on the subject related to its effect on health, work safety, and hearing. Past researchers studied construction workers and found that training only had a small impact on PPE use [4]. Studies have also found that the responsibility lies with the employers to take the initiative and motivate their workers to use HPD, especially in noisy work environments. Employers’ training on safety measures is vital to increase their employees’ knowledge and competence related to safety measures while performing their duties [60]. Intervention development at the organizational level, such as policy development, will also assist in motivating employees to use HPD in a noisy area, thereby minimizing the risk of NIHL [31].

A study examined the factors influencing Thai workers’ use of HPDs, namely organizational factors, such as company rules and regulations, provision of hearing protection devices, dissemination of knowledge and information, noise monitoring, and hearing testing [31]. Organizational support is also a significant variable in predicting HPD use among firefighters [37]. The researchers recommended that employers prescribe and enforce rules and regulations related to HPD use to ensure full compliance. Nevertheless, the association between organizational support and HPD use changed from positive to negative upon introducing interpersonal influences in the model built using regression analysis. A positive correlation between organizational support and HPD use was more accurate in representing the relationship between the variables, as the collinearity between both variables was not statistically significant [37].

### 4.4. Cognitive-Perceptual Factor

Perceived barrier. Previous studies have reported various reasons for workers’ unwillingness to use HPDs, such as their inability to communicate when using HPDs, feelings of discomfort, and bulky and inconvenient equipment [1,35,36,45,48]. Specifically, past researchers found that reluctance to use HPD was due to discomfort experienced by workers attributed to heat, humidity, and communication difficulties. Some studies found external ear canal asymmetry does not significantly influence personal attenuation rating (PAR) [1]. In addition, workers have often complained that PPE use affects their productivity and reduces their work efficiency [37]. Surprisingly, some workers believed that wearing HPDs compromises their safety and causes infections due to unhygienic earplugs [27]. This view is shared by farmers who claim that hearing protectors do not protect them from hearing loss [32]. Other studies reported that discomfort was the main barrier or obstacle preventing workers from using HPD [31,41,43]. 

Apart from discomfort, previous studies regarding perceived barriers suggested that most workers do not wear HPDs due to their bulky nature, thereby causing substantial logistical and physical inconvenience [1,35,36,45,48]. These issues could be overcome by providing workers with information regarding the benefits (particularly its safety aspect) of HPD use and the importance of offering choices in selecting HPDs for industrial workers. This could be performed via training programs emphasizing the purpose and benefits of wearing HPD and methods to reduce discomfort. In addition, training could help workers wear HPD properly [61]. Therefore, training performed either individually or in small groups would significantly benefit the participants [62]. 

Perceived susceptibility. A past study reported that perceived susceptibility to hearing loss was a significant predictor of hearing protection behavior among firefighters [37]. Addressing perceived susceptibility will motivate workers to wear HPDs in order to consistently reduce the risk of hearing loss. These findings corroborate the reports of a study where perceived susceptibility was positively associated with PPE compliance [49]. Meanwhile, other studies also found that perceived susceptibility influenced the levels of HPD use among workers [37,49]. Awareness of excessive noise exposure effects is important to increase the worker’s risk perception. Therefore, a hearing conservation program (HCP) is an important tool introduced by employers to benefit their workers and increase safety levels at the workplace [63]. However, the risks associated with excessive noise exposure cannot be minimized. 

Perceived severity. Workers’ perception of the workplace’s physical strain influences their HPD use [28]. There was a correlation between HPD use and perceived hearing loss among construction workers [30]. It was also reported that perceived severity is a positive predictor of PPE compliance among wastewater workers [49]. Therefore, the perceived severity factor must be considered when developing safety programs and interventions to enhance PPE compliance. This finding is consistent with the study by Thepaksorn et al. [47] that examined HPD use among sawmill workers. Scholars have highlighted the importance of risk perception and knowledge, which must be factored into the design and implementation of safety intervention programs among workers. Therefore, risk perception is the main predictor for HPD use [47,48]. Individual risk perception is the most significant predictor of HPD use [28]. Earlier studies demonstrated that perceived severity risks on the use of HPD do influence employees to wear them consistently. Hence, a noise risk assessment is vital to identify and measure noise exposure at the workplace, including personal noise monitoring, area of noise monitoring, and audiometry tests as stated under the Occupational Safety and Health (Noise Exposure) Regulations 2019 [63]. Noise exposure levels also influenced HPD use among workers [33]. 

Perceived benefit. Reducing the annoying effects of noise is an incentive for workers to use HPD. The workers’ perceived benefit of using HPD to protect their hearing is a vital part of life [36]. Perceived hearing status was significantly associated with the use of HPDs among workers [45]. Perceived benefits emerged as the strongest predictor of HPD use, which is consistent with the reports by [34,37]. Wearing HPD considerably reduced sound levels compared to not wearing them [64]. The researchers also found that masked thresholds were considerably lower when HPD was worn at 85 dBA compared to no HPD use. These findings suggested that HPD could fulfill its intended function. 

Furthermore, using HPD increased the amount of listening effort required [65]. In a noisy work environment, this increased listening effort resulted in cognitive fatigue. Therefore, the perceived benefit among workers could be enhanced if the workers understand the advantages of wearing the HPD. This is in line with a statement by a study where increased knowledge among people may be translated into how people think and act [66].

Self-efficacy. Self-efficacy affects HPD use among workers [34,37]. Among the factors influencing HPD use, perceived self-efficacy was the most important factor compared with perceived benefit and perceived barriers [34]. Although self-efficacy and benefits of HPD use reflected high scores in surveys conducted by earlier researchers, the use of HPDs among farmers was low, especially if they perceived high barriers [32]. 

Workers’ perceived benefit and self-efficacy will trigger them to be consistent in wearing the hearing protector [32]. Nevertheless, McCullagh et al. [32] also found that these two cognitive-perceptual factors render them ineffective if the worker’s perceived barrier is high. Therefore, employers must increase the frequency of their training programs to help provide positive information regarding the benefits of using a hearing protector. Training programs resulted in positive behavioral change among workers [29]. Effective training will also increase knowledge of the significance of protecting one’s auditory faculty [36]. Additionally, education and health promotion behaviors in relation to PPE use are crucial in minimizing occupational hazards at the workplace. Overall, these events will increase workers’ perceived benefits and self-efficacy toward PPE compliance [49].

### 4.5. Health-Promoting Behaviour

Cues to action. Health-promoting behavior is a goal or action that aims to achieve a favorable health outcome, such as optimal well-being, personal fulfillment, and productive life [67]. Cognitive-perceptual factors influence health-promoting behavior [68]. The subthemes of cues to action are factors that appear to be a cue, or a trigger, for taking suitable action. The combined degree of susceptibility and severity provided the energy or force to act, and the perception of rewards (fewer barriers) offered a preferred way of action [69]. The cues to action originating from the Health Belief Model could play a crucial role in reducing workers’ occupational noise exposure and protecting their health [49]. Wastewater workers across the Southeast Region of the US reported that cues to action were advantageous in wearing PPE. Posters such as reminders, frequent education on the importance of wearing PPE, availability of PPE at the hazard area, seeing others wearing PPE, and supervision by the supervisor enhance workers’ compliance with PPE regulations [49]. 

The cues to action are also necessary to increase PPE compliance [49]. Disciplinary action accompanied by incentives and education is crucial to encourage workers to adopt healthy and safety-compliant behaviors. Earlier studies have suggested the significance of PPE enforcement among workers [70]. The best way to improve PPE compliance is by providing relevant information to workers through a short video, statistics, and posting reminders [71]. A study reported that the construct of cues to action under the Health Belief Model would improve compliance to standard precaution (SP) by providing reminders and education on SP procedure, thereby triggering a change in the behavior of people [69,72]. The additional factor of cues to action in the Health Belief Model should be considered in future research to overcome the limitations of the current model. A review found that the cues to action influence perceived susceptibility and perceived severity in predicting behavioral change [73]. The cues to action also have a direct implication on the use of PPE [74]. Other than personal conviction, environmental influences impacted one’s decision to wear PPE. Family, society, the media, and the government all play essential roles in encouraging people to adopt preventative steps [75]. Statistically, PPE was shown to be 2.4 times more likely to be worn by workers who were more attentive to environmental cues [76].

### 4.6. Future Directions and Recommendations

This study has bridged the gaps in understanding the factors influencing the use of HPDs. Thus, the variables derived from this study could contribute new knowledge for future scholarly work. This type of review could overcome the issues facing industries in encouraging workers to wear HPDs. The findings would assist industrial owners in understanding the significant factors that could encourage workers to consistently wear HPDs in a noisy work environment. Then, we hope that this systematic study offers significant points of reference for upcoming researchers as well as valuable input to expert practitioners and scholars in a variety of industries. This study suggested several recommendations for the consideration of future scholars.

Utilizing various databases such as Cochrane Library, DOAJ, My Jurnal, and Research Gate could widen the search and retrieval of relevant articles, thereby generating more data to support the study objectives. Future studies should focus more on cues to action towards human behavior on the use of HPD. This factor could assist in triggering and compelling industrial workers to take suitable action in wearing the HPD. Workers’ health status and comorbidities of hearing loss need to be considered under sociodemographic characteristics, such as blood glucose level, hearing disorder, psychosocial health, cardiovascular disease, cancer, stroke, and vertigo. These factors may lead to industrial workers’ action in using HPD. Finally, as industrial workers gain more knowledge regarding noise and noise-related disease, their cognitive-perceptual factors theme could be influenced in using the HPD consistently.

## 5. Conclusions

This SLR investigated the factors influencing HPD use between 2006 and 2021. Five themes emerged from this study, namely, sociodemographic (29%), interpersonal influences (18%), situational influences (18%), cognitive-perceptual (29%), and health-promoting behavior (6%). Additionally, 17 sub-themes emerged by systematically reviewing the 28 articles from Scopus, Web of Sciences, Science Direct, PubMed, Wiley Online Library, and Google Scholar databases following the PRISMA guidelines. The use of HPD among industrial workers was found to be multifactorial and impossible to reduce to a single factor. Conclusively, the significant factors influencing workers to use HPDs are sociodemographic, interpersonal influences, situational influences, and health-promoting behavior. The cues to action factor under the health-promoting behavior could sufficiently trigger the worker’s behavior to take protective action by wearing the HPD while working in a noisy work area. Moreover, the intense stimuli from these cues to action would be needed to trigger a response. Thus, the cues to action could be a very useful factor in making workers consistently wear the HPD while working in a noisy work environment.

## Figures and Tables

**Table 1:** The search string.

Database	Search String
Scopus	TITLE-ABS-KEY ((“hearing protection device*” OR “hearing protector*” OR “earplug*” OR “ear muff*” OR “ear protector*” OR “hearing protection*”) AND (“factor*” OR “aspect*” OR “consideration*” OR “cause*”) AND (“industrial worker*” OR “manufacturing worker*” OR “industrial employer*” OR “industrial people*” OR “industrial group*” OR “construction worker*”))
Web of Science	TS = ((“hearing protection device*” OR “hearing protector*” OR “earplug*” OR “ear muff*” OR “ear protector*” OR “hearing protection*”) AND (“factor*” OR “aspect*” OR “consideration*” OR “cause*”) AND (“industrial worker*” OR “manufacturing worker*” OR “industrial employee*” OR “industrial people*” OR “industrial group*” OR “construction worker*”))

**Table 2:** Inclusion and exclusion criteria.

Criterion	Eligibility	Exclusion
Literature type	Journal and Conference proceeding (research article)	Journal (systematic review), book series, book, chapter in the book
Language	English	Non-English
Timeline	Between 2006–2021	<2006

**Table 3:** Themes and sub-themes.

Authors	Ref.	Country	1. Sociodemographic					2. Interpersonal Influences			3. Situational Influences			4. Cognitive Perceptual Factor					5. Health Promoting Behaviour
			A	G	EL	NL	WE	SM	IS	SN	SC	T	OS	PB	PS	PV	BF	SE	CA
Hong et al. (2006)	[26]	United States				/			/				/	/	/		/		
Robertson et al. (2007)	[27]	United States		/				/						/	/				
Arezes and Miguel (2008)	[28]	Portugal									/					/			
Neitzel et al. (2008)	[29]	United States										/							
Edelson et al. (2009)	[30]	USA			/						/					/			
Tantranont et al. (2009)	[31]	Thailand						/		/				/					
McCullagh et al. (2010)	[32]	USA		/										/				/	
Sbihi et al. (2010)	[33]	Canada	/	/															
Kim et al. (2010)	[34]	USA				/		/						/				/	
Seixas et al. (2011)	[4]	USA							/	/		/							
Ahmed (2012)	[35]	UAE												/					
Reddy et al. (2012)	[36]	New Zealand						/		/		/		/			/		
Hong et al. (2013)	[37]	USA											/		/			/	
Bockstael et al. (2013)	[2]	Belgium				/	/				/								
Dutra et al. (2015)	[38]	USA									/								
Meira et al. (2015)	[39]	Brazil		/				/											
Lu et al. (2015)	[40]	China						/		/									
Taban et al. (2016)	[41]	Iran	/	/	/	/	/							/					
Mccullagh et al. (2016)	[42]	USA	/							/		/							
Jeffree et al. (2016)	[43]	Malaysia												/					
Gongi et al. (2016)	[44]	Kenya							/										
Tantranont and Codchanak (2017)	[45]	Thailand							/					/			/		
Feder et al. (2017)	[46]	Canada				/	/												
Thepaksorn et al. (2018)	[47]	Thailand										/				/			
Tinoco et al. (2019)	[48]	Brazil	/	/								/		/		/			
Cavallari et al. (2019)	[15]	United States									/								
Wright et al. (2019)	[49]	USA													/	/			/
Copelli et al. (2021)	[1]	Canada												/					
**Sociodemographic**		**Interpersonal Influences**				**Situational Influences**					**Cognitive-Perceptual**					**Health Promoting Behaviour**			
A = Age G = Gender EL = Educational Level NL = Noise Level WE = Working Experience		SM = Social Models IS = Interpersonal Support SN = Social Norms				SC = Safety Climate T = Training OS = Organisational Support					PB = Perceived Barrier PS = Perceived Susceptibility PV = Perceived Severity PF = Perceived Benefit SE = Self-Efficacy					CA = Cues to Action			

## Data Availability

The data availability statement is not applicable to this study.
